# Complete chloroplast genome of an invasive marine macroalga *Ulva californica* (Ulvophyceae, Chlorophyta)

**DOI:** 10.1080/23802359.2022.2098854

**Published:** 2022-07-22

**Authors:** Xuyin Lin, Wenzheng Liu, Xiu Wei, Peng Jiang

**Affiliations:** a School of Marine Biology, Xiamen Ocean Vocational College, Xiamen, China; b CAS and Shandong Province Key Laboratory of Experimental Marine Biology, Center for Ocean Mega-Science, Institute of Oceanology, Chinese Academy of Sciences, Qingdao, China; c Laboratory for Marine Biology and Biotechnology, Qingdao National Laboratory for Marine Science and Technology, Qingdao, China; d University of Chinese Academy of Sciences, Beijing, China; e College of Life Science, Qingdao University, Qingdao, China

**Keywords:** Chloroplast genome, invasive species, phylogenetic analysis, *Ulva californica*

## Abstract

Species belonging to *Ulva* (Ulvophyceae, Chlorophyta) are one of the major members of invasive seaweeds. *Ulva californica* Wille 1899 was originally believed to be native to the Pacific coast of North America, while in recent years it has been reported as exotic species, or new record, in Europe, the Mediterranean, Asia, and Oceania. However, the paths of global dispersal of *U. californica* are unclear. In addition, the species boundary between *U. californica* and a related species is somewhat disputed. Here, we reported that the complete chloroplast genome of *U. californica* is 92,126 bp in size, harboring 96 genes (GenBank accession no. MZ561475). The overall base composition was A (37.9%), T (37.4%), C (12.3%), and G (12.4%), similar to those from other *Ulva* species. The phylogenomic analysis showed that although *U. californica* was genetically closer to *Ulva aragoënsis* (Bliding) Maggs 2018 in [Krupnik N et al., 2018], they were clearly distinguishable, supporting the recent opinion that they should be separated into different species. The chloroplast genome data of *U. californica* would provide plenty resources for phylogeography analysis and monitor on bioinvasion.

Due to global warming and a variety of anthropogenic activities such as mariculture and discharge of ballast water, macroalgae can spread or be introduced into new habitats, and species in the genus *Ulva* (Ulvophyceae, Chlorophyta) are one of the major members (Verlaque and Breton 2019; Xie et al. 2020; Liu et al. 2022). *Ulva californica* Wille 1899 was initially described with the type location at La Jolla, California (Collins et al. 1899), and two other taxa, i.e. *U. angusta* Setchell & N.L. Gardner 1920 and *U. scagelii* Chihara 1969, were later placed into the synonymy with it (Tanner 1986). *U. californica* was originally believed to be native to the Pacific coast of North America (Scagel et al. 1989; Wolf et al. 2012), but some speculate that it may be more widespread (Loughnane et al. 2008). Nevertheless, in recent years *U. californica* has been reported as exotic species, or new record, in Europe (Hayden and Waaland 2004; Loughnane et al. 2008), the Mediterranean (Wolf et al. 2012; Sfriso et al. 2020), Asia (Kawai et al. 2007), and Oceania (Heesch et al. 2009; Kirkendale et al. 2013), even rapid local spreads after introduction have been observed in Germany (Steinhagen et al. 2019), and China (Wei et al. 2022). However, the paths of global dispersal of *U. californica* are unclear. In addition, the species boundary between *U. californica* and a related species is somewhat disputed. According to results of molecular identification and hybridization examination, *U. mediterranea* Alongi, Cormaci & G.Furnari 2014, which was later revised to *U. aragoënsis* (Bliding) Maggs 2018 in [Krupnik N et al., 2018] (Krupnik et al. 2018), was distinguished from *U. californica* and *U. flexuosa* Wulfen 1803 (Hiraoka et al. 2017), while in a later study these species were still combined into one complex (Steinhagen et al. 2019). Organelle genome data from *U. californica* can help clarify this controversy, and provide sufficient molecular markers to reveal geographic origins and dispersal routes.

Here, we sequenced the chloroplast genome of *U. californica* sample U484-3, which was collected from Putian, Fujian Province, China in 2021 (25°12′17″N, 119°33′51″E), and cultured in laboratory with Von Stosch’s Enriched (VSE) medium at 16 °C under a light intensity of 80–100 μmol·m^−2^·s^−1^. This kind of plant study did not need specific permissions from the ethics committee of Institute of Oceanology, Chinese Academy of Sciences (IOCAS), and the field collection was carried out following the National standards of the People's Republic of China (2007). A specimen was deposited in Marine Biological Museum of Chinese Academy of Sciences (MBMCAS) at IOCAS (http://www.qdio.cas.cn, Yongqiang Wang, bmxia@qdio.ac.cn) under the voucher number MBM287040.

The algal tissue was sent to BENAGEN Co. Ltd. (Wuhan, China) for high-throughput sequencing. Total genomic DNA was extracted using a Plant Genomic DNA Extraction Kit (Tiangen Biotech Co., Ltd., Beijing, China). The library of genomic DNA was sequenced using the Illumina and Nanopore platform. The read length for Illumina was 150 bp. The total amount and base of reads were 30,479,638 and 4.6 Gbp for Illumina, and 1,149,830 and 5.4 Gbp for Nanopore platform, respectively. A short sequence assembly software Flye v.2.8.3 was used to assemble clean data (Kolmogorov et al. 2020) and the obtained complete chloroplast genome sequence was annotated with PGA (Qu et al. 2019).

The complete chloroplast genome of *U. californica* was 92,126 bp in size (GenBank accession no. MZ561475). The overall base composition was A (37.9%), T (37.4%), C (12.3%), G (12.4%), and the percentage of AT (75.3%) is much higher than CG (24.7%), which were similar to those from other *Ulva* species. This genome encodes 96 genes, including 68 protein-coding genes, 26 transfer RNAs genes, and two ribosomal RNAs genes. Using MEGA 6.0 with a GTR + G+I model (Tamura et al. 2013), a maximum-likelihood (ML) phylogenetic tree was constructed with 24 complete chloroplast genomes of *Ulva* and one chloroplast genome of *Pseudoneochloris marina* as the outgroup. It was shown that, although *U. californica* was genetically closer to *Ulva aragoënsis* (Cai et al. 2017), they were clearly distinguishable (Figure 1), supporting the recent opinion that they should be separated into different species (Hiraoka et al. 2017). The data of *U. californica* chloroplast genomes can be used as resources for phylogeography analysis and monitor on bioinvasion, even risk of green tides dominated by this species (Bae 2010).

**Figure 1.**
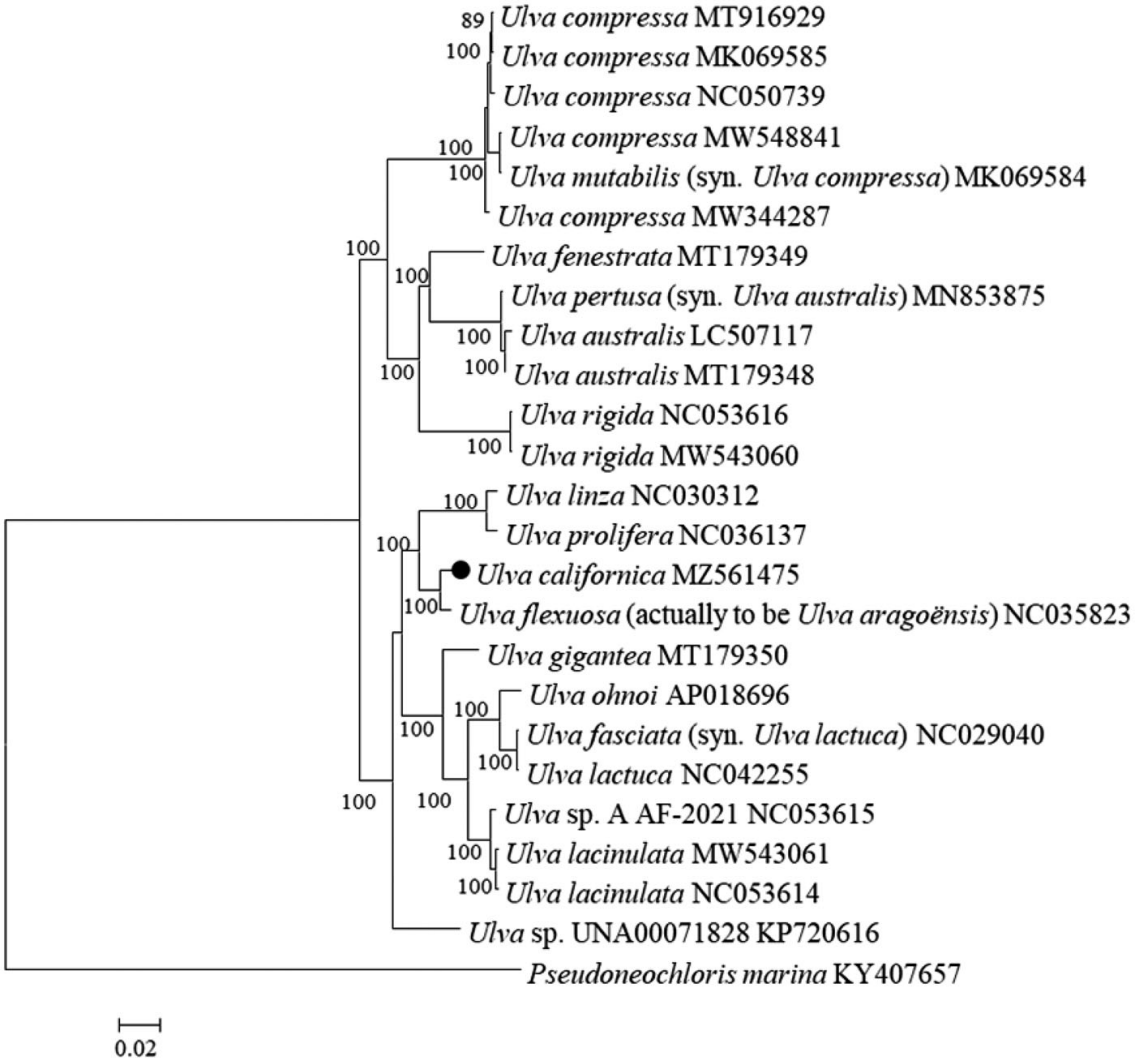
Phylogenetic tree based on maximum-likelihood (ML) analysis with 24 *Ulva* chloroplast genomes and one chloroplast genome from *Pseudoneochloris marina* as the outgroup. Numbers above each node indicate the bootstrap support value. The black dot represents the sequence from the sample used in this study.

## Data Availability

The genome sequence data that support the findings of this study are openly available in GenBank of NCBI at https://www.ncbi.nlm.nih.gov under the accession no. MZ561475. The associated BioProject, SRA, and Bio-Sample numbers are PRJNA788066, SRR17206483, and SAMN23896768, respectively.

## References

[CIT0001] Bae EH. 2010. Ulotrichales, Ulvales. In: Bae EH, Kim HS, Kwon CJ, Hwang IK, Kim GH, Klochkova TA, editors. Algal flora of Korea. Incheon, Korea: National Institute of Biological Resources; p. 7–52.

[CIT0002] Cai CE, Wang LK, Zhou LJ, He PM, Jiao BH. 2017. Complete chloroplast genome of green tide algae *Ulva flexuosa* (Ulvophyceae, Chlorophyta) with comparative analysis. PLOS One. 12(9):e0184196. 28863197 10.1371/journal.pone.0184196 PMC5581003

[CIT0003] Collins FS, Holden I, Setchell WA. 1899. Phycotheca Boreali-Americana. A collection of dried specimens of the algae of North America. Malden (MA).

[CIT0004] Hayden HS, Waaland JR. 2004. A molecular systematic study of *Ulva* (Ulvaceae, Ulvales) from the northeast Pacific. Phycologia. 43(4):364–382.

[CIT0005] Heesch S, Broom JES, Neill KF, Farr TJ, Dalen JL, Nelson WA. 2009. *Ulva*, *Umbraulva* and *Gemina*: genetic survey of New Zealand taxa reveals diversity and introduced species. Eur J Phycol. 44(2):143–154.

[CIT0006] Hiraoka M, Ichihara K, Zhu WR, Shimada S, Oka N, Cui JJ, Tsubaki S, He PM. 2017. Examination of species delimitation of ambiguous DNA-based *Ulva* (Ulvophyceae, Chlorophyta) clades by culturing and hybridisation. Phycologia. 56(5):517–532.

[CIT0007] Kawai H, Shimada S, Hanyuda T, Suzuki T, Gamagori City Office. 2007. Species diversity and seasonal changes of dominant *Ulva* species (Ulvales, Ulvophyceae) in Mikawa Bay, Japan, deduced from ITS2 rDNA region sequences. Algae. 22(3):221–228.

[CIT0008] Kirkendale L, Saunders GW, Winberg P. 2013. A molecular survey of *Ulva* (Chlorophyta) in temperate Australia reveals enhanced levels of cosmopolitanism. J Phycol. 49(1):69–81. 27008390 10.1111/jpy.12016

[CIT0009] Kolmogorov M, Bickhart DM, Behsaz B, Gurevich A, Rayko M, Shin SB, Kuhn K, Yuan J, Polevikov E, Smith TPL, et al. 2020. MetaFlye: scalable long-read metagenome assembly using repeat graphs. Nat Methods. 17(11):1103–1110. 33020656 10.1038/s41592-020-00971-x PMC10699202

[CIT0010] Krupnik N, Rinkevich B, Paz G, Douek J, Lewinsohn E, Israel A, Carmel N, Mineur F, Maggs CA. 2018. Native, invasive and cryptogenic *Ulva* species from the Israeli Mediterranean Sea: risk and potential. Mediterranean Mar Sci. 19(1):132–146.

[CIT0011] Liu JL, Tong YC, Xia J, Sun YQ, Zhao XH, Sun JY, Zhao S, Zhuang MM, Zhang JH, He PM. 2022. *Ulva* macroalgae within local aquaculture ponds along the estuary of Dagu River, Jiaozhou Bay, Qingdao. Mar Pollut Bull. 174:113243. 34920239 10.1016/j.marpolbul.2021.113243

[CIT0012] Loughnane CJ, McIvor LM, Rindi F, Stengel DB, Guiry MD. 2008. Morphology, *rbc*L phylogeny and distribution of distromatic *Ulva* (Ulvophyceae, Chlorophyta) in Ireland and Southern Britain. Phycologia. 47(4):416–429.

[CIT0013] National Standards of the People's Republic of China. 2007. Specifications for oceanographic survey. Part 6: marine biological survey. GB/T 12763.6.

[CIT0014] Qu XJ, Moore MJ, Li DZ, Yi TS. 2019. PGA: a software package for rapid, accurate, and flexible batch annotation of plastomes. Plant Methods. 15:50. 31139240 10.1186/s13007-019-0435-7 PMC6528300

[CIT0015] Scagel RF, Gabrielson PW, Garbary DJ, Golden L, Hawkes MW, Lindstrom SC, Oliveira JC, Widdowson TB. 1989. A synopsis of the benthic marine algae of British Columbia, Southeast Alaska, Washington and Oregon. Canada: Phycological Contributions, University of British Columbia; p. 532.

[CIT0016] Sfriso A, Buosi A, Wolf MA, Sfriso AA. 2020. Invasion of alien macroalgae in the Venice Lagoon, a pest or a resource? Aquat Invasions. 15(2):245–270.

[CIT0017] Steinhagen S, Karez R, Weinberger F. 2019. Cryptic, alien and lost species: molecular diversity of *Ulva sensu lato* along the German coasts of the North and Baltic Seas. Eur J Phycol. 54(3):466–483.

[CIT0018] Tamura K, Stecher G, Peterson D, Filipski A, Kumar S. 2013. MEGA6: molecular evolutionary genetics analysis version 6.0. Mol Biol Evol. 30(12):2725–2729. 24132122 10.1093/molbev/mst197 PMC3840312

[CIT0019] Tanner CE. 1986. Investigations of the taxonomy and morphological variation of *Ulva* (Chlorophyta): *Ulva californica* Wille. Phycologia. 25(4):510–520.

[CIT0020] Verlaque M, Breton G. 2019. Biological invasion: long term monitoring of the macroalgal flora of a major European harbor complex. Mar Pollut Bull. 143:228–241. 31789158 10.1016/j.marpolbul.2019.04.038

[CIT0021] Wei X, Liu WZ, Lin XY, Liu QC, Jiang P. 2022. First record of Ulva californica in mainland China: a single alien parthenogenetic population with discontinuous distribution. J Oceanol Limnol.

[CIT0022] Wolf MA, Sciuto K, Andreoli C, Moro I. 2012. *Ulva* (Chlorophyta, Ulvales) biodiversity in the North Adriatic Sea (Mediterranean, Italy): cryptic species and new introductions. J Phycol. 48(6):1510–1521. 27010000 10.1111/jpy.12005

[CIT0023] Xie WF, Wu CH, Zhao J, Lin XY, Jiang P. 2020. New records of *Ulva* spp. (Ulvophyceae, Chlorophyta) in China, with special reference to an unusual morphology of *U. meridionalis* forming green tides. Eur J Phycol. 55(4):412–425.

